# EZH2 promotes DNA replication by stabilizing interaction of POLδ and PCNA via methylation-mediated PCNA trimerization

**DOI:** 10.1186/s13072-018-0213-1

**Published:** 2018-08-02

**Authors:** Peng A, Xinyi Xu, Chenglin Wang, Jing Yang, Shida Wang, Jiewen Dai, Ling Ye

**Affiliations:** 1 State Key Laboratory of Oral Diseases, West China School of Stomatology, Sichuan University, Chengdu, People’s Republic of China; 2 Shanghai Ninth People’s Hospital, Shanghai Jiao Tong University School of Medicine, Shanghai, People’s Republic of China

**Keywords:** EZH2, PCNA, DNA replication, Methylation

## Abstract

**Background:**

Proliferating cell nuclear antigen (PCNA), a ring-shaped homotrimer complex, promotes DNA replication via binding to DNA polymerase. Trimerized PCNA is critical for DNA replication. Enhancer of zeste homologue 2 (EZH2), which primarily acts as a histone methyltransferase, is essential for proliferation. However, how EZH2 promotes proliferation by controlling DNA replication through PCNA remains elusive.

**Results:**

Here, we showed that low EZH2 levels repressed the proliferation of human dental pulp cells (hDPCs). The EZH2 protein level was dramatically upregulated in hDPCs at S phase in the absence of H3K27 trimethylation. Molecularly, EZH2 interacted with PCNA via the PIP box and dimethylated PCNA at lysine 110. Dimethylation of PCNA is essential for stabilization of the PCNA trimer and the binding of DNA polymerase δ to PCNA.

**Conclusions:**

Our data reveal the direct interaction between PCNA and EZH2 and a novel mechanism by which EZH2 orchestrates genome duplication.

**Electronic supplementary material:**

The online version of this article (10.1186/s13072-018-0213-1) contains supplementary material, which is available to authorized users.

## Background

Replication of the genome is an essential step in cell cycle progression and proliferation. Failure in this process will lead to abnormal cell proliferation, cell cycle arrest, and genomic instability [1–3]. At the heart of DNA replication, proliferating cell nuclear antigen (PCNA) has an essential role in orchestrating normal DNA synthesis by providing a platform to which DNA polymerases and other factors bind via the PIP box [4]. PCNA, a ring-shaped sliding clamp, encircles DNA by the dimerization (prokaryotes) or trimerization (eukaryotes) of monomers [5–7]. Post-translational modifications (PTMs) of PCNA facilitate these protein interactions and are essential for the high processivity and accuracy of DNA synthesis. Modifications on PCNA, carried by “writers,” profoundly regulate the interaction between PCNA and its partners. Phosphorylation, acetylation, ubiquitination, and sumoylation on PCNA have been strongly suggested to regulate DNA replication by affecting the binding affinity of PCNA partners or the stability of PCNA [8–11]. Additionally, SETD8, a methyltransferase, has been reported to interact with, and methylate PCNA [12, 13].

Protein lysine methylation has been widely elucidated in histone modification [14, 15]. However, accumulating non-histone proteins have been reported to be methylated by histone methyltransferases [16, 17]. Enhancer of zeste homologue 2 (EZH2), a methyltransferase, is primarily responsible for the trimethylation of histone H3 lysine 27 (H3K27me3) [18–21]. In addition, EZH2 has been reported to methylate non-histone proteins, such as cardiac transcription factor 4 (GATA4), retinoic acid-related orphan nuclear receptor α (RORα), and signal transducer and activator of transcription 3 (STAT3) [22–24]. EZH2 is positively related to proliferation, and its role as a transcriptional silencer via repression of INK4A/ARF is well established [25–27]. Beyond its role as the transcriptional repressor, EZH2 was also reported to localize at the replication fork by interacting with histone in response to DNA damage [28, 29]. Moreover, a recent study suggests that Polycomb (PcG) repressive complexes (PRCs) control proliferation independent of transcriptional repression of cell cycle-regulating genes [30]. However, there is still a lack of evidence indicating whether EZH2 directly binds to PCNA, and information regarding whether EZH2 participates in DNA replication remains elusive.

In this study, we demonstrated, for the first time, that EZH2 directly binds to PCNA via the PIP box and promotes methylation at lysine 110. Then, we showed that K110me2 is critical for the stabilization of the PCNA trimer. Finally, we showed that EZH2 promotes the interaction between DNA polymerase δ (POLδ) and PCNA.

## Results

### EZH2 regulates HDPC proliferation independent of its role as a gene repressor

**Fig. 1:**
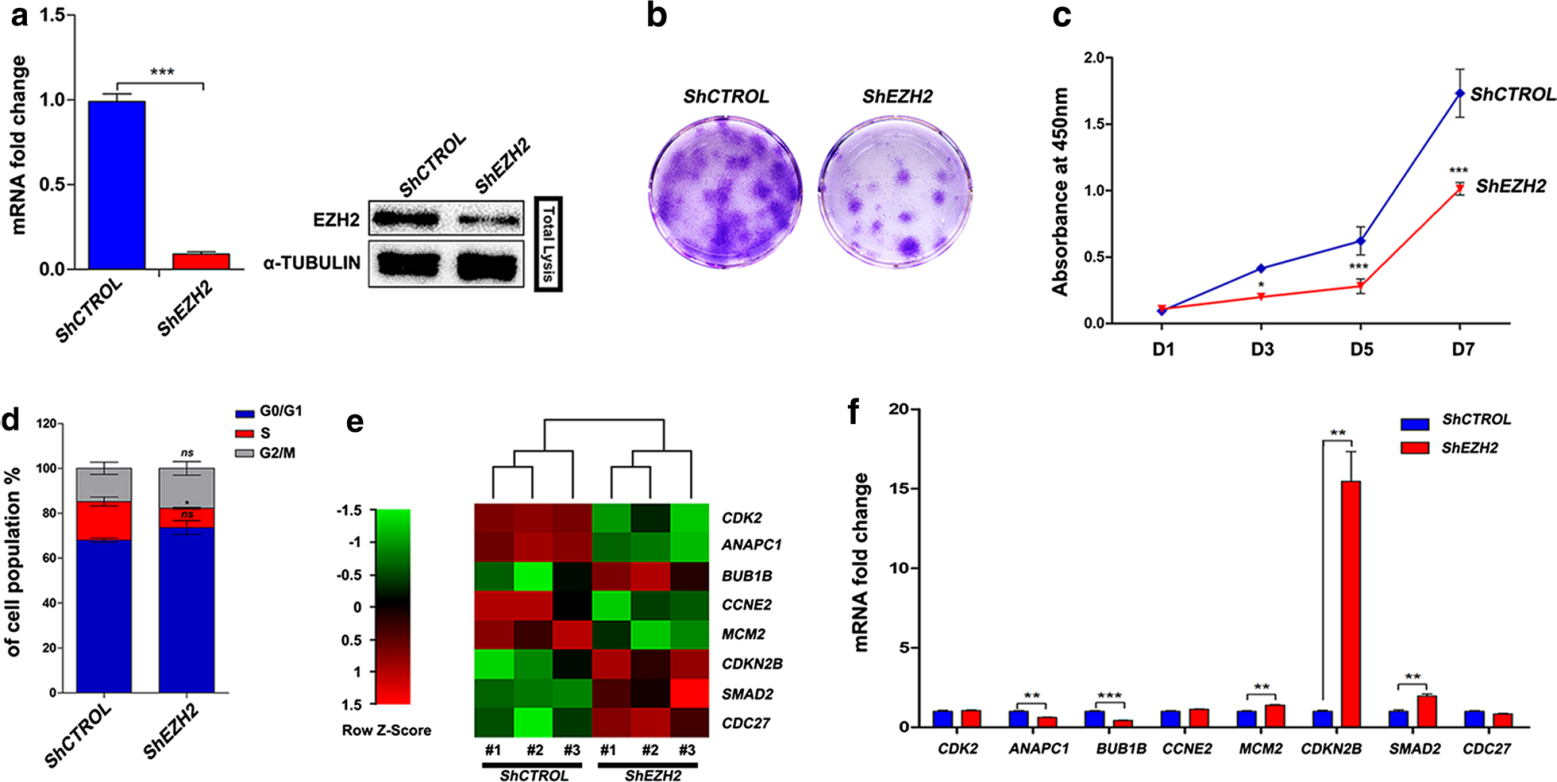
EZH2-knockdown hDPCs show decreased proliferation, but no transcriptionally significant changes are observed in DNA replication-related genes. **a** Quantitative PCR and western blot show EZH2 knockdown with shRNA in hDPCs (72 h after transduction). **b** Colony formation assay by crystal violet staining demonstrates the viability of EZH2 knockdown hDPCs. **c** Growth curves measured with CCK-8. **d** Flow cytometry analysis shows the cell cycle distribution of EZH2-knockdown hDPCs. **e** Cell cycle RNA-sequencing assay shows the transcripts with significantly altered expression in EZH2-knockdown hDPCs. **f** Quantitative PCR verifies the expression levels of genes identified in the RNA-Seq data. (*ns* not significant, *P < 0.05, **P < 0.01, ***P < 0.001)

EZH2 is highly expressed in actively proliferating cells and controls cell proliferation by regulating cell cycle checkpoint signalling via histone methyltransferase activity [25, 26]. First, we investigated how EZH2 influences proliferation through the regulation of checkpoint signalling in hDPCs. shRNA was used to knock down the expression of EZH2 in hDPCs (Fig. 1a). We observed that hDPC proliferation was strongly impaired upon EZH2 knockdown as indicated by the results of the colony formation assay and growth curves (Fig. 1b, c). Cells exhibited a reduction in the percentage of cells in S phase. Interestingly, no significant increase in the percentage of cells in either G0/G1 phase or G2/M phase was observed (Fig. 1d). Our observation was contrary to previous studies in which EZH2 depletion led to an increase in the percentage of cells in G2/M phase [31, 32].

To experimentally validate these observations, the expression of cell cycle-related genes was measured using a cell cycle RNA-sequencing assay in EZH2-knockdown hDPCs from three different donors. EZH2 has been reported to function as a transcriptional repressor [21] and represses the expression of *INK4A, INK4B, and ARF* [27, 33]. In the RNA-sequencing analysis of EZH2-knockdown hDPCs, eight genes were transcriptionally altered (Fig. 1e); however, there was no transcriptional difference in *INK4A* and *ARF*. Among the eight genes, cyclin-dependent kinase 2 (*CDK2*), cyclin E2 (*CCNE2*), and cyclin-dependent kinase inhibitor 2B (*CDKN2B,* also known as *INK4B*) are involved in controlling cell cycle phase transition [26, 34]. *CDK2* and *CCNE2*, which also regulate the DNA replication process, were downregulated, and *CDKN2B* was upregulated. Interestingly, when verifying the differentially expressed genes in the RNA-Seq data by qPCR in hDPCs from a fourth donor, we found that only *ANAPC1, CDKN2B,* and *SMAD2* exhibited the same changes in gene expression (Fig. 1f). These findings are consistent with a previous study showing that Polycomb (PcG) controls the proliferation of mouse embryonic fibroblasts (MEFs) independent of Ink4a/Arf suppression [30].

Combined with the observation that the defect in proliferation upon EZH2 knockdown did not elicit significant cell cycle arrest at G1 or G2/M phase, our data suggest that instead of canonically controlling transcription at cell cycle phase transitions [27], EZH2 may regulate the proliferation of hDPCs with an alternative mechanism (Additional file 1: Figure S1).

### EZH2 elevates at S phase in hDPCs and interacts with PCNA

**Fig. 2:**
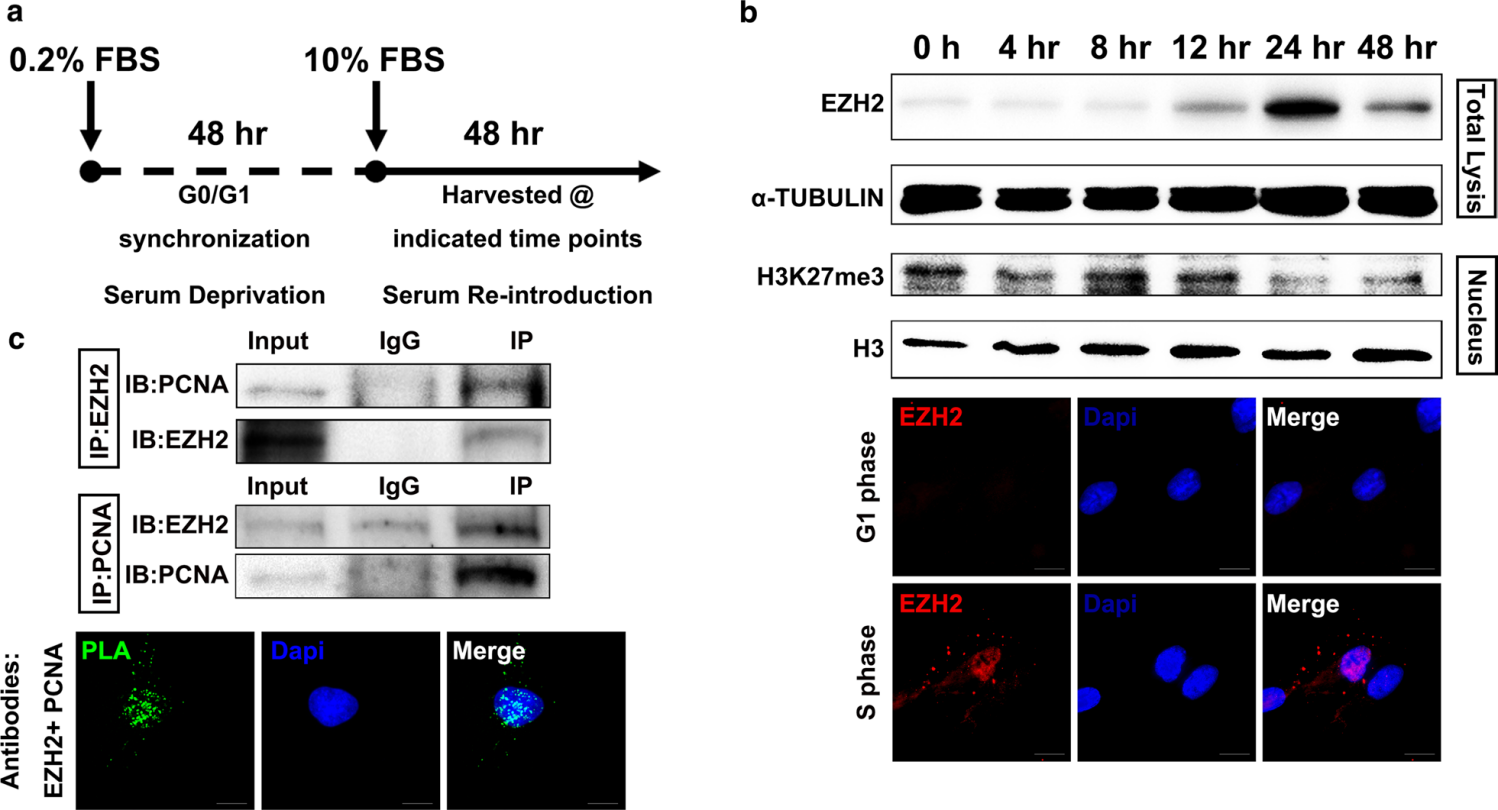
EZH2 elevates at S phase in hDPCs and interacts with PCNA. **a** Schematic chart illustrating the serum deprivation approach used for G0/G1 phase synchronization of hDPCs. **b** Western blot shows the expression of EZH2 in hDPCs at the indicated time points after release from serum deprivation. Immunofluorescence shows the subcellular localization of EZH2 in hDPCs in G1 or S phase. **c** Co-immunoprecipitation (Co-IP) of EZH2 and PCNA in hDPCs (synchronized at S phase) and proximity ligation assay (PLA) after co-incubating anti-EZH2 and anti-PCNA antibodies in hDPCs in S phase. IgGs were used as control antibodies for the IP. Antibodies used for IP and western blot are labelled as IP and IB, respectively. Total lysate (10 μg) was used as an input control. Scale bars represent 20 μm

The proliferation of hDPCs was independent of EZH2 transcriptional activity (Fig. 1). In addition, a previous study showed that PRCs control the proliferation of MEFs instead of functioning in their traditional role as a transcriptional repressor [30]. Thus, we hypothesized that EZH2 could be directly involved in events during S phase. To investigate this, we first examined the expression dynamics of EZH2 in the cell cycle of hDPCs. hDPCs were synchronized at G0 phase by serum deprivation for 48 h and subsequently released from deprivation by the addition of 10% FBS to allow entry into the cell cycle (Fig. 2a). Then, cells were harvested at the indicated time points and subjected to flow cytometry to examine the cell cycle distribution (Additional file 2: Figure S2A); furthermore, the protein level of EZH2 was investigated at the indicated time points. We found that EZH2 protein accumulated at S phase (24 h); however, the levels of trimethylated histone H3 lysine 27 (H3K27me3) were decreased at S phase (Fig. 2b). Recently, studies reported a lower accumulation of H3K27me3 following DNA replication [35, 36], which is consistent with our observations. Immunofluorescence was performed at 8 and 24 h, which correspond to G1 phase and S phase, respectively. Consistently, compared to the levels in G1 phase, EZH2 protein levels were increased and had accumulated in the nucleus during S phase (Fig. 2b, Additional file 2: Figure S2C left), and the accumulation was further confirmed by Pearson’s correlation coefficient analysis of EZH2 and DAPI staining (Additional file 2: Figure S2C, right). These data indicated a new role for EZH2 in the biological process of S phase independent of catalysing H3K27 trimethylation.

Because cells duplicate their genome during S phase, we hypothesized that EZH2 might participate in the DNA replication of hDPCs in S phase. PCNA, a sliding clamp, is at the heart of DNA synthesis by providing the platform for factors to orchestrate DNA replication [4, 37]. Additionally, EZH2 has been reported to be present at the replication fork in human cells [29, 38]. Thus, we investigated whether EZH2 directly interacts with PCNA in hDPCs. We performed co-immunoprecipitation (co-IP) of endogenous EZH2 and PCNA (Fig. 2c, upper panel). Moreover, by using a proximity ligation assay (PLA) with EZH2- and PCNA-specific antibodies, we visualized the interaction of EZH2 and PCNA at the molecular level (Fig. 2c, lower panel). Additionally, the interaction of EZH2 and PCNA was verified in 293T cells (data not shown). These results indicate that EZH2 directly interacts with PCNA and strongly indicates the participation of EZH2 in DNA replication.

### The presence of a PIP box in EZH2

**Fig. 3:**
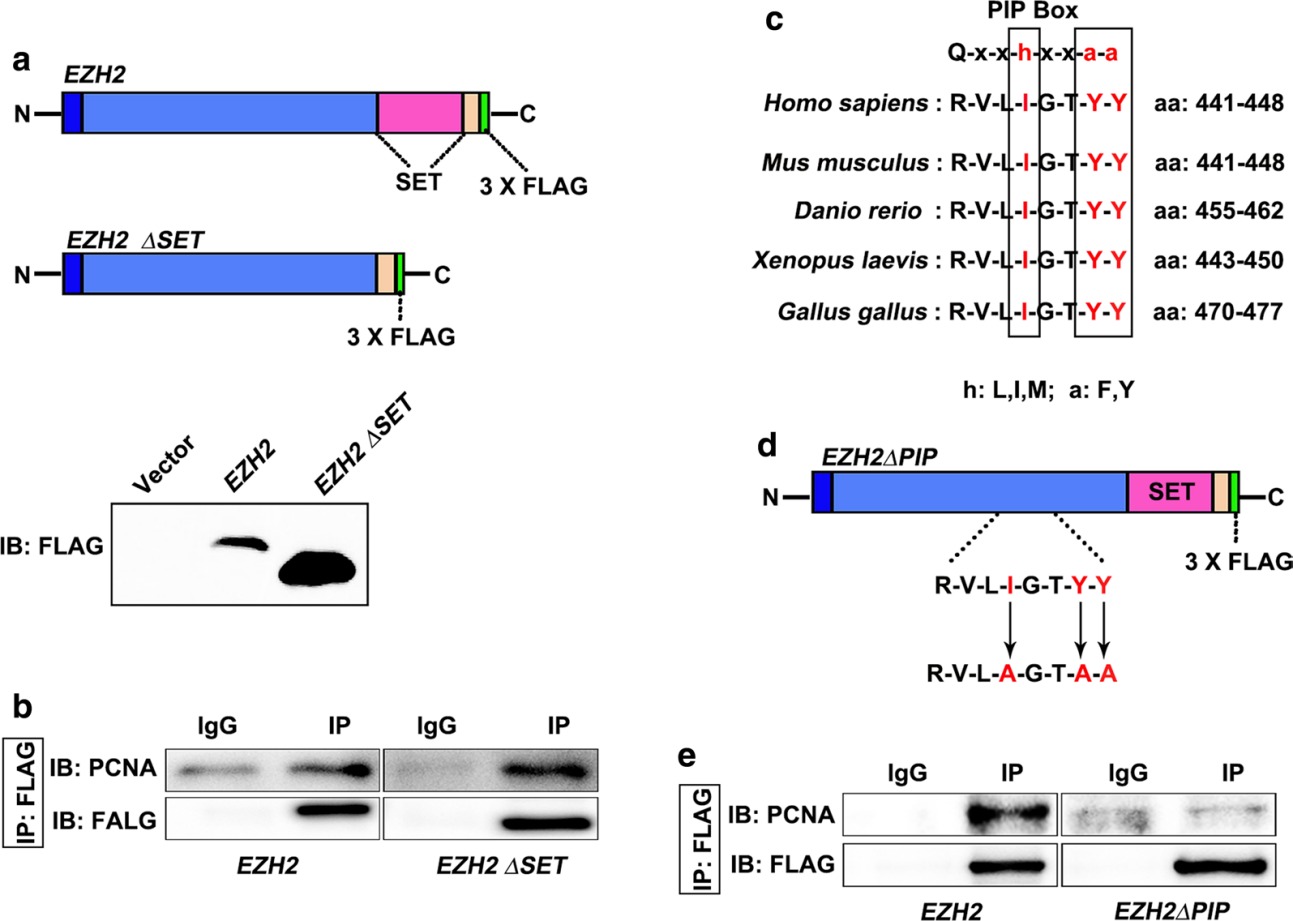
Presence of a PIP box in EZH2. **a** Schematic representation of FLAG-tagged full-length EZH2 (*EZH2*) and FLAG-tagged SET-domain deletion mutation EZH2 *(EZH2∆SET)*. The lower panel shows the expression of EZH2 in *EZH2*- and *EZH2∆SET*-transfected 293T cells. **b** IP illustrates the binding of PCNA to EZH2 in *EZH2*- and *EZH2∆SET*-transfected 293T cells. **c** The sequence and location of the PIP box in EZH2 homologues of different species. Conserved amino acids are highlighted: *h* represents a hydrophobic amino acid (L/I/M), *a* represents an aromatic (*F*/*Y*), and *x* represents any amino acid. *aa* amino acid. **d** Schematic representation of the full-length EZH2 with mutations of conserved PIP box amino acids (*EZH2∆PIP*). **e** IP shows the interaction of EZH2 and PCNA in *EZH2∆PIP* 293T cells. IgGs were used as control antibodies for IP. Antibodies used for IP and western blot are labelled as IP and IB, respectively

SET (SU [VAR] 3–9, Enhancer of Zeste, Trithorax)-domain proteins usually bind to their catalytic substrates via the SET domain [39–41]. To investigate whether the binding of EZH2 to PCNA also occurs in a SET domain-dependent manner, we transfected 293T cells with *EZH2∆SET* (SET domain-truncated mutation) (Fig. 3a) and then examined the interaction between EZH2 and PCNA using IP in wild-type *EZH2*- or *EZH2∆SET*-transfected 293T cells. Interestingly, deletion of the SET domain showed no effect on the ability of PCNA to bind EZH2 (Fig. 3b), suggesting that the interaction between EZH2 and PCNA is governed by other mechanisms rather than the binding of the SET domain. PCNA has been reported to interact with other proteins via the evolutionally conserved PIP box [4, 42] or the AlkB homologue 2 PCNA-interacting motif (APIM) [43] present in those proteins. Additionally, histone methyltransferases have also been reported to possess a PIP box motif to interact with PCNA [12, 44]. Thus, we assessed whether EZH2 contains either a putative PIP box or the APIM. When surveying the amino acid sequence of EZH2, we found a conserved putative PIP box (RVL*I*GT*YY*) (Fig. 3c) but not the APIM motif. To determine whether EZH2 interacts with PCNA via the putative PIP box, we mutated the conserved amino acid residues isoleucine (*I*) and tyrosine (*Y*) in the PIP box to alanine (*A*) residues (*EZH2∆PIP*) (Fig. 3d). Then, we performed IP with a FLAG-tagged antibody in 293T cells transfected with wild-type *EZH2* or *EZH2∆PIP*. PCNA was precipitated in cells transfected with wild-type EZH2 but not in cells transfected with EZH2∆PIP (Fig. 3e). Taken together, these results strongly suggest that EZH2 is a novel PCNA-interacting protein and possesses an evolutionarily conserved PIP box.

### EZH2 methylates PCNA

**Fig. 4:**
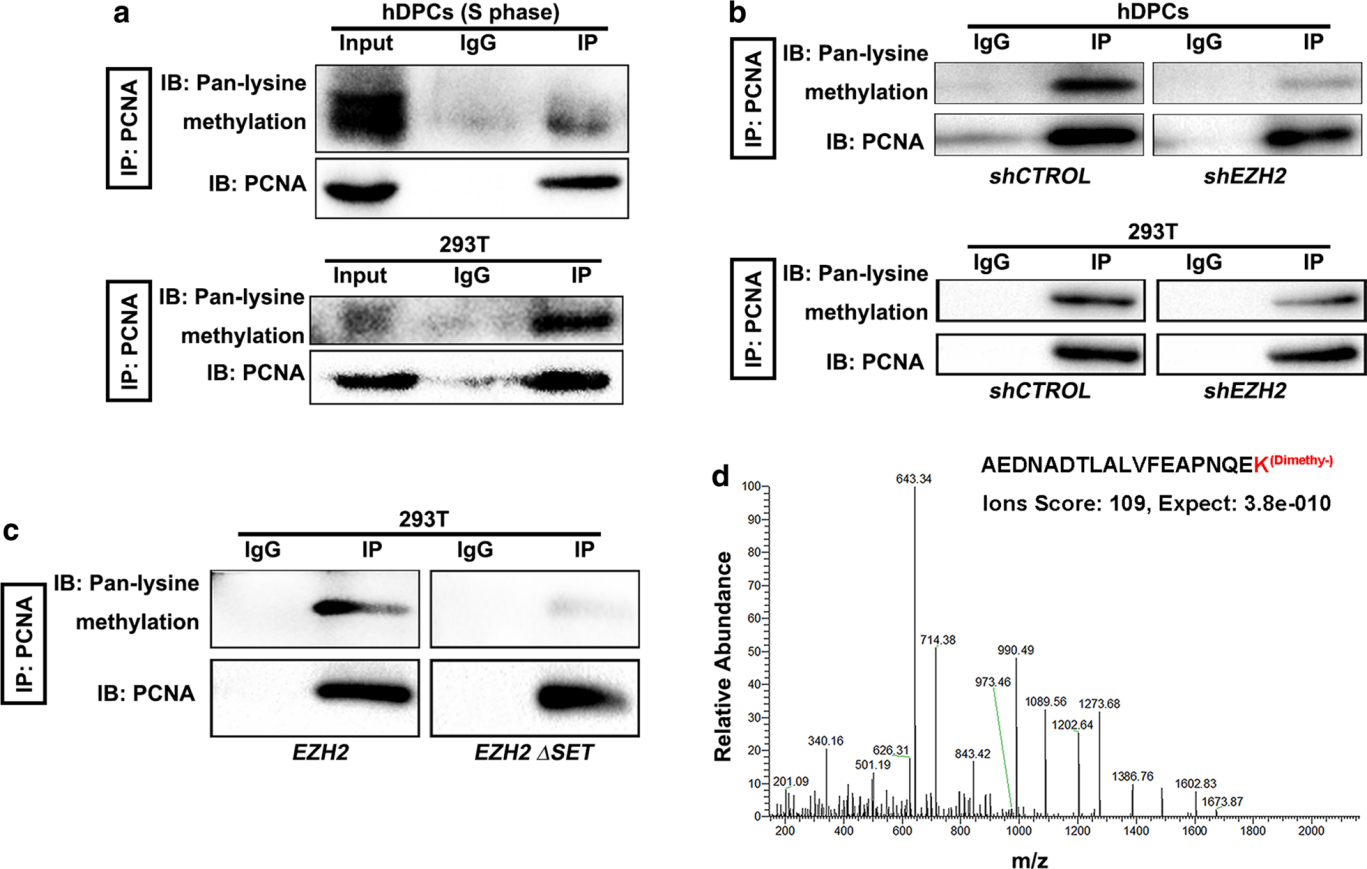
Methylation of PCNA by EZH2. **a** Pan-lysine methylation of PCNA in hDPCs (S phase) and 293T cells was demonstrated with IP and IB. **b** IP shows the pan-lysine methylation status of PCNA after knocking down EZH2. **c** The pan-lysine methylation of PCNA after overexpressing *EZH2* or *EZH2 ∆SET* in 293T cells. **d** Mass spectrometry analysis of trypsin-digested PCNA by LC–MS/MS. PCNA proteins were immunoprecipitated with anti-PCNA antibody from EZH2-overexpressing 293T cells. The tandem mass-fragmentation spectrum represents the PCNA peptide containing residues 92–110. A mass shift of + 28 was observed. IgGs were used as control antibodies for IP. Antibodies used for IP and western blot are labelled as IP and IB, respectively. Total lysate (10 μg) was used as an input control

As a histone lysine methyltransferase, EZH2 promotes the addition of mono-, di- or trimethyl groups onto its histone substrates [18–20, 45]. Moreover, EZH2 has recently been reported to interact with non-histone proteins and to promote the methylation of non-histone proteins [22–24]. Additionally, because our data showed that EZH2 directly interacts with PCNA (Fig. 2), we hypothesize that PCNA might be a catalytic substrate of EZH2. To evaluate this, we first tested the methylation status of PCNA in hDPCs and 293T cells. Using IP with an anti-PCNA antibody in hDPCs and 293T cells followed by immunoblot with a pan-methyl lysine antibody, we found the presence of methylated lysines on PCNA in both hDPCs and 293T cells (Fig. 4a). Next, to investigate the role of EZH2 in PCNA methylation, we performed IP with a PCNA antibody in EZH2-knockdown hDPCs or 293T cells and then examined the methylation status of PCNA. In both cases, PCNA methylation was significantly decreased (Fig. 4b). These data suggested that EZH2 is required for the lysine methylation of PCNA. To further determine whether the methyltransferase activity of EZH2 is necessary for PCNA methylation, we assessed the methylation status of endogenous PCNA in *EZH2∆SET*-transfected 293T cells. Compared to wild-type EZH2, *EZH2∆SET*-transfected 293T cells exhibited markedly decreased methylation of PCNA (Fig. 4c). Furthermore, to investigate the site at which EZH2 promoted methylation on PCNA, we overexpressed EZH2 in 293T cells, immunoprecipitated endogenous PCNA with an anti-PCNA antibody, and subjected the PCNA gel band to mass spectrometry analysis. A dimethyl modification was identified at the lysine (110) residue of PCNA (Fig. 4d). Taken together, our data suggested that PCNA is a catalytic substrate of EZH2 and is dimethylated at lysine 110.

### Stabilized trimerization of PCNA by EZH2 is essential for the binding of DNA polymerase δ to PCNA

**Fig. 5:**
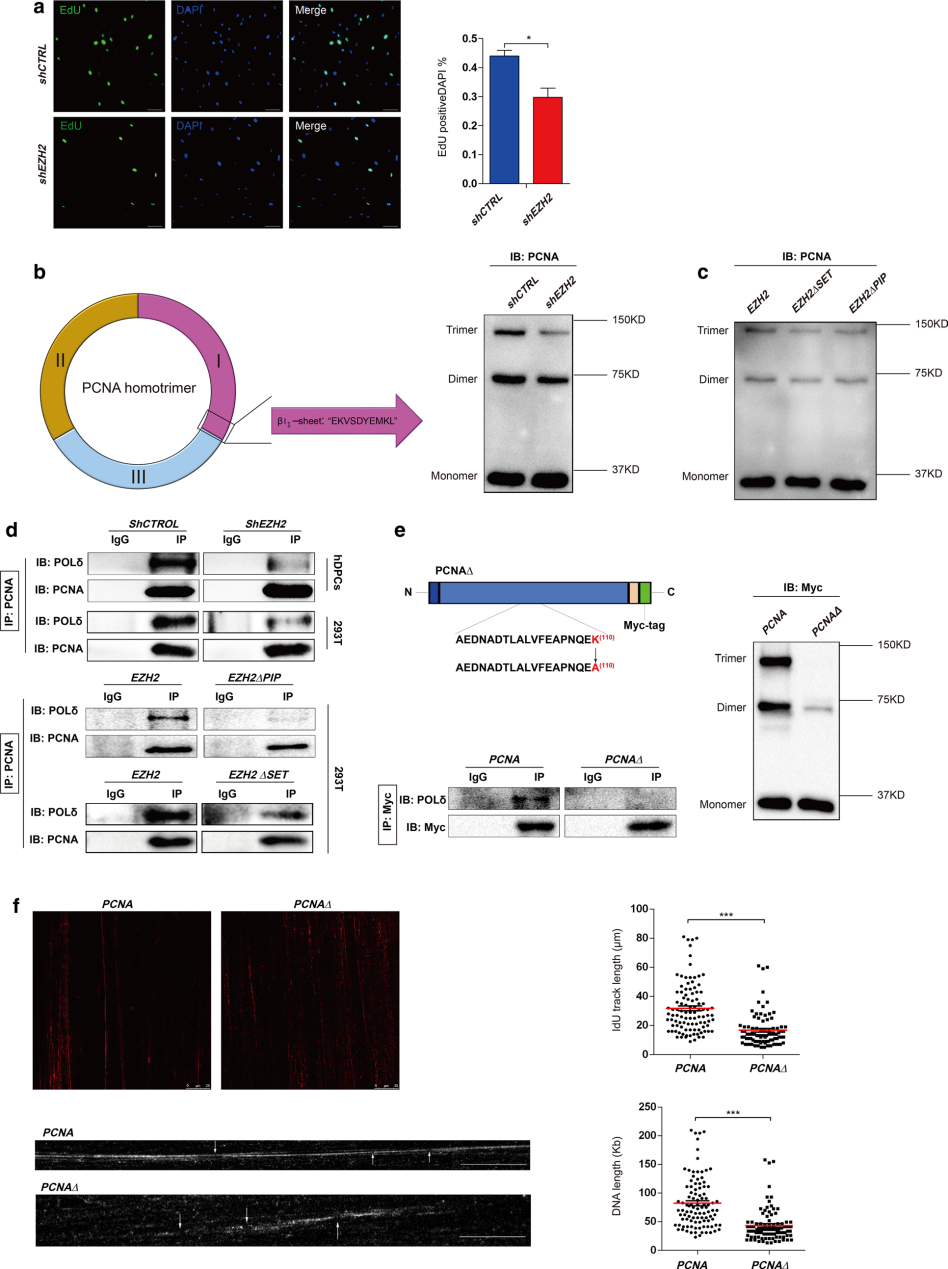
Methylation of PCNA at lysine 110 affects the binding of DNA polymerase δ to PCNA. **a** A 3 h EdU pulse was applied in EZH2-knockdown hDPCs at 21 h after release from serum deprivation. Scale bars represent 100 μm. **b** Formaldehyde protein crosslinking assay indicates the presence of the trimer form of PCNA in EZH2-depleted 293T cells. The left panel illustrates the location of lysine 110 on the “head” β-sheet of the interface between the homo-monomers. **c** Crosslink assay shows the level of the trimer form of PCNA in 293T cells overexpressing *EZH2∆SET* and *EZH2∆PIP*. **d** IP demonstrates the decreased interaction between PCNA and POLδ when knocking down EZH2 or overexpressing *EZH2∆PIP* and *EZH2∆SET* in 293T cells. **e** Schematic representation of full-length PCNA with a point mutation at lysine 110 (K110). A Myc tag was added on the C-terminus. The crosslink assay shows the level of trimer PCNA upon lysine 110 mutation (*PCNA∆*). IP demonstrates the binding of POLδ to PCNA when *PCNA∆* is overexpressed in 293T cells. **f** DNA fibre assay demonstrates DNA synthesis in cells expressing the lysine 110 mutant (*PCNA∆*). Origins are indicated by arrows. Scatter plot and bar chart show the DNA length (***P < 0.001). IgGs were used as a control antibody for IPs. Antibodies used for IP and western blot are labelled as IP and IB, respectively

As a scaffold for DNA polymerases, PCNA plays a crucial role in DNA synthesis [37, 46]. Thus, we next investigated how EZH2 regulates DNA replication in hDPCs. First, we conducted an EdU pulse assay for 3 h in EZH2-knockdown hDPCs. We found that fewer EZH2-knockdown hDPCs cells were actively synthesizing DNA than wild-type cells, as illustrated by the reduced EdU incorporation (Fig. 5a). PCNA, a ring-shaped clamp, consists of three homo-monomers arranged in a “head-to-tail” manner in eukaryotes [5–7, 47]. Upon further exploration of the structure of PCNA, we identified that the methylated lysine residue is located on the β-sheet of the PCNA monomer where two monomers meet to establish the “head-to-tail” complex. We therefore hypothesized that EZH2 could regulate the stabilization of the trimer form of PCNA. We then applied a protein crosslink strategy to evaluate the stabilization of the PCNA trimers. EZH2 depletion reduced the formation of the PCNA trimer (Fig. 5b). We used the same crosslink strategy to investigate whether stabilization of the PCNA trimer was dependent on the protein methylation capacity of EZH2. Stabilization of the PCNA trimer was impaired in cells expressing either the SET domain-truncated mutation or the PIP box mutation (Fig. 5c). Taken together, our results indicate that EZH2 is essential for the stabilization of the PCNA trimer.

In normal DNA replication, enhancing the processivity of DNA polymerase δ (POLδ) is the primary function of PCNA [46, 48, 49]. Thus, we explored whether EZH2 could affect the binding of POLδ to PCNA. First, we immunoprecipitated endogenous PCNA protein using a PCNA antibody to assess the interaction between POLδ (represented by the catalytic subunit, POLD1) and PCNA in hDPCs and 293T cells with EZH2 knockdown. We found decreased binding of POLD1 to PCNA when EZH2 was knocked down (Fig. 5d). We next investigated whether the binding of POLδ to PCNA depends on the interaction between EZH2 and PCNA by examining the interaction between PCNA and POLδ in *EZH2∆PIP*-transfected 293T cells. We found that the binding of POLD1 to PCNA was dramatically reduced upon mutation of the PIP box (Fig. 5d). Moreover, we also investigated the effect of the methyltransferase activity of EZH2 on the interaction between PCNA and POLδ. Compared to the cells expressing wild-type *EZH2*, cells transfected with *EZH2 ∆SET* showed decreased levels of POLD1 precipitated with PCNA (Fig. 5d). Taken together, these results indicate that the methyltransferase activity of EZH2 is necessary for the interaction between PCNA and POLδ.

As our data demonstrated that PCNA can undergo EZH2-mediated dimethylation at lysine 110, we hypothesized that K110me2 may affect the stabilization of the PCNA trimer. We introduced a point mutation at lysine 110 on PCNA (Fig. 5e), expressed this mutant in 293T cells, and then conducted the protein crosslink assay. As demonstrated, the trimer form of PCNA was dramatically impaired upon mutating PCNA at this residue (Fig. 5e). Moreover, the binding of POLδ to PCNA was dramatically impaired (Fig. 5e). Based on the fact that K110A-mutated PCNA exhibited impaired binding to POLδ, we next tested whether this mutant PCNA could affect DNA synthesis. By using a DNA fibre assay, we observed that the DNA length dramatically decreased in cells expressing PCNA with the K110A mutation (Fig. 5f).

Taken together, our data suggested that the K110me2 catalysed by EZH2 is important for the trimerization of PCNA and the binding of POLδ to PCNA.

## Discussion

Epigenetic modulators such as methyltransferases have been widely studied in the histone lysine methylation field with regard to their modulation of chromatin properties and regulation of gene expression [21, 50–52]. However, owing to growing interest in the post-translational modifications of proteins, the importance of methylation on non-histone proteins has frequently been indicated, and non-histone proteins have been reported to be potential substrates of histone lysine methyltransferases [16, 17]. In the present study, we demonstrated that PCNA is a novel substrate of EZH2 that can be dimethylated at K110. To date, only three non-histone proteins, RORα, GATA4, and STAT3, have been reported to be substrates of EZH2 [22–24]. In addition, lysine methylation is reversible, and H3K27 has been shown to be demethylated by KDM6B [15, 53]. Thus, the occurrence of demethylation on PCNA and the verification of de-methyltransferase activity on PCNA need to be elucidated in future. Recently, studies have reported a lower accumulation of H3K27me3 following DNA replication [35, 36], which is consistent with our observation of decreased H3K27me3 in hDPCs in S phase.

In the present study, we found that knockdown of EZH2 did not induce a significant cell cycle arrest at G1 or G2/M phase, but the proliferation activity of hDPCs was continuously repressed. This was also supported by the observation that the transcript levels of most cell cycle regulatory genes did not change when EZH2 was knocked down in hDPCs. These findings are consistent with previous studies showing that Polycomb (PcG) controls the proliferation of MEFs independent of INK4A/ARF suppression [30].

PCNA, a sliding clamp, plays a key role in duplicating the genome by providing a scaffold for relevant factors and DNA polymerases. PCNA interacts with DNA replication cofactors during DNA replication via the evolutionarily conserved PIP box or APIM motif [4, 43, 54]. Previous studies reported that the SET-domain proteins ATXR5 and ATXR6, which have primarily been demonstrated to be H3K27 mono-methyltransferases, interacted with PCNA in Arabidopsis [44, 55]. In addition, other epigenetic modifiers, such as DNMT1, HDAC1, p300, and SETD8, were reported to interact with PCNA in humans [11, 13, 56, 57]. So far, SETD8 is the only histone methyltransferase that has been reported to methylate PCNA [13]. Furthermore, previous studies suggested that in response to DNA damage, EZH2 was recruited to stalled replication forks to function as an H3K27me3 methyltransferase [28, 29]. However, there was no evidence indicating a direct interaction between EZH2 and PCNA. Here, we provide the first evidence that EZH2 directly interacts with PCNA via the conserved PIP box motif to promote the methylation of PCNA. In the study by Andrea Piunti et al., the association between RING1B and PCNA is reduced in the absence of EZH2, suggesting the possible hierarchical recruitment of PRC1 and PRC2 [30, 58]. In our study, we confirmed the interaction between EZH2 and PCNA; thus, we hypothesized that the reduced association between RING1B and PCNA was possibly influenced by the diminished interaction between EZH2 and PCNA when EZH2 was depleted. However, this mechanism needs to be verified in future.

PCNA, a ring-shaped complex, consists of three homo-monomers that assemble in a “head-to-tail” manner in eukaryotes [4]. Maintenance of the PCNA trimer is a prerequisite for promoting the processivity of DNA polymerase δ (POLδ). Here, we uncovered that PCNA K110me2 catalysed by EZH2 is essential for stabilizing the trimer form of PCNA. Moreover, consistent with previous studies [30, 59], we observed an interaction between SUZ12 and PCNA (data not shown). This suggested that other PRC2 components might be involved in the regulation of PCNA methylation. The trimerization of PCNA is established through the binding of β-sheets of each monomer at the interfaces between the subunits [5, 6, 60]. Three types of interactions between the monomer interfaces of PCNA have been proposed to stabilize the PCNA trimer: hydrogen bonds, hydrophobic interactions, and ion pairs. Among these, ion pairs between the β-sheets and the interface were considered the main contributor [60]. Based on the investigation of PCNA among various species, a positive polar amino acid, lysine, was found at position 110, which is located within the β-sheet [60]. Here, we report that K110me2 is critical for the trimerization of PCNA. Methylation does not perturb the charge of the lysine residue and induces a small change in its size; thus, K110me2 is more likely to enhance the hydrophobic interaction between the monomers to stabilize the PCNA trimer by changing the hydrogen property and hydrophobic force of lysine 110 [17, 60, 61]. To verify this hypothesis, further structural studies are needed. Moreover, in normal DNA replication, PCNA interacts with POLδ and stimulates its processing activity [4, 47, 62, 63]. Here, we claimed that EZH2-mediated dimethylation of PCNA K110 is essential for the interaction of PCNA with POLδ, leading to the control of DNA synthesis. Because EZH2 regulates the trimerization of PCNA, it is evident that the binding of POLδ to PCNA decreases in the absence of EZH2.

## Conclusion

**Fig. 6:**
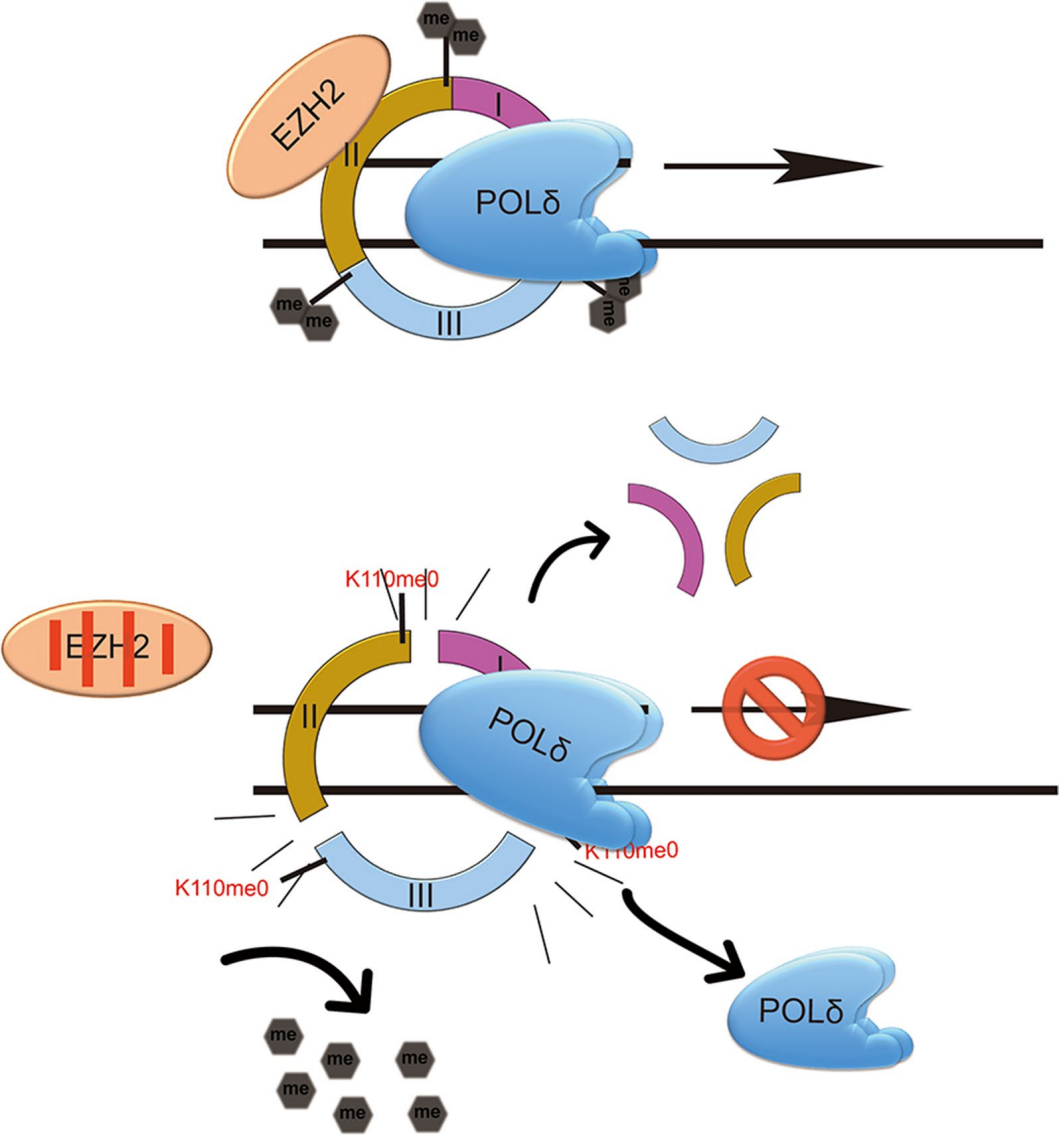
Predicted model by which EZH2 regulates DNA replication via dimethylation of PCNA. In normal cells, EZH2-mediated dimethylation of PCNA at K110 is required for the DNA replication performed by POLδ. Upon repression of the transcription or the methyltransferase activity of EZH2 or inhibition of the interaction between EZH2 and PCNA, stabilization of the PCNA trimer is perturbed, resulting in a defect in POLδ binding

In conclusion, our results provide novel evidence that PCNA is a novel lysine methylation substrate of EZH2 and that EZH2 is a new PCNA-interacting protein. EZH2-dependent dimethylation of PCNA K110 is essential for DNA replication because it affects the binding of POLδ to PCNA via stabilization of the PCNA trimer (Fig. 6). There experiments reveal a novel mechanism through which epigenetic regulators participate and control DNA replication.

## Methods

### Cell culture

hDPCs were harvested from normal impacted third molars extracted from adult humans (19–22 years old) in West China Hospital of Stomatology as described previously [64]. The procedures were approved by the Ethical Committee of the West China School of Stomatology, Sichuan University, and performed in accordance with approved guidelines; all subjects provided informed consent. HEK293T (ATCC) cells were obtained from Ohio (Shanghai). In all experiments, cells were grown in DMEM (GIBCO) supplemented with 10% FBS (GIBCO) and 1% antibiotics (GIBCO). HEK293T cells were cultured in growth medium on plates coated with 0.01% poly-l-lysine (Sigma-Aldrich). All cells were grown at 37 °C in a humidified atmosphere containing 5% CO_2_.

For G0/G1 synchronization, hDPCs were grown in 0.2% FBS-containing medium for 48 h, after which they were cultured in normal growth medium and harvested at the indicated time points.

### Lentivirus production and transduction

Lentivirus expressing *shEZH2* (forward oligo: 5′-CCGG*GAGGGAAAGTGTATGATAA*CTCGAG*TTATCATACACTTTCCCTC*TTTTTG-3′, reverse oligo: 5′-AATTCAAAAA*GAGGGAAAGTGTATGATAA*CTCGAG*TTATCATACACTTTCCCTC***-**3′) was generated by inserting shRNA oligos against *EZH2* into the pLKO.1-TRC cloning vector (gifts from Zhipeng Fan, Capital Medical University) following the Addgene protocol. Oligos were synthesized by Invitrogen. The control vector was also a gift from Zhipeng Fan. Constructs containing wild-type *EZH2*, *EZH2∆SET*, PIP box point mutations of EZH2 (*EZH2∆PIP*), wild-type *PCNA*, or point mutations of PCNA (*PCNA∆*) were acquired from Ohio (Shanghai). Lentiviruses containing shRNA were produced in 293T cells with the following plasmids: 9 µg of pLKO.1 shRNA vector, 6 µg of *∆*8.2 packaging plasmid, and 3 µg of VSV-G envelope plasmid. After 60 h, the supernatant containing viral particles was harvested and filtered at 0.45 μm and added to either hDPCs or 293T cells in the presence of 5 μg/ml polybrene (Sigma-Aldrich) for 48 h. Then, positive cells were selected with 2 μg/ml puromycin (Sigma-Aldrich) for 3 days, and the cells were maintained in growth medium containing 1 μg/ml puromycin.

### Western blot analysis

For immunoblots, cells were lysed in protein lysis solution (Pierce Biotechnology) containing protease inhibitor cocktail (Millipore) and phosphatase inhibitor cocktails (Millipore). Protein extraction was performed according to the manufacturer’s instructions. For nuclear protein detection, a nuclear extraction procedure was applied following the manufacturer’s protocol (Pierce Biotechnology). After the proteins were subjected to standard SDS–polyacrylamide gel (SDS-PAGE) electrophoresis (the percentages were based on the molecular weight of proteins of interest), they were transferred to nitrocellulose membranes (Millipore), which were blocked for 1 h at room temperature in TBST (0.1% Tween-20) containing either 5% skim milk or bovine serum albumin (Sigma-Aldrich). Then, the membranes were incubated overnight at 4 °C with primary antibodies diluted in blocking buffer, washed 5X with TBST, and incubated for 1 h in secondary antibodies diluted in blocking buffer (Table 1). Membranes were washed with Restore Western Blot Stripping Buffer (Thermo) following the manufacturer’s protocol. Next, the membranes were washed 5X with TBST, and enhanced chemiluminescence reagent (Millipore) was added to the membranes. Proteins were visualized using ImageLab (Bio-Rad) according to the manufacturer’s instructions (Additional file 3: Figure S3).

**Table 1:** Antibodies used in this study

Antibody	Brand	Cat no.	Use	Condition
EZH2	BD	612666	WB, IF	1:2000, 1:200
EZH2 (AC22)	Merck Millipore	17-662	IP	2/500 μg
EZH2	R&D	AF4767	PLA	1:50
Histone H3	Abcam	ab24834	WB	1:1000
α-Tubulin	Abcam	ab52866	WB, IF	1:5000, 1:500
H3K27me3(C36B11)	Cell signalling	9733	WB	1:2000
PCNA (PC10)	Cell signalling	2586	WB, IF, PLA, IP	1:5000, 1:200
Pan-methyl lysine	Abcam	ab7315	WB	1:2000
DNA Polymerase delta (607)	Abcam	ab10362	WB	1:1000
CYCLIN A2	Abcam	ab38	WB	1:500
CYCLIN D1	Abcam	ab40754	WB	1:5000
FLAG-tag	Sigma	F7425	WB, IP	1:2000, 1:100
Myc-Tag	Cell signalling	2278	WB, IP	1:4000, 1:200
Ubiquityl-PCNA (Lys164) (D5C7P)	Cell signalling	13439	WB	1:1000
Histone H2AX	Abcam	Ab11175	WB	1:2500
γ-H2A.X	Cell signalling	9718	WB	1:2000
Goat anti-rabbit IgG (HRP)	Abcam	ab6721	WB	1:10,000
Goat anti-mouse IgG (HRP)	Abcam	ab6789	WB	1:10,000
Rabbit anti-goat IgG-FITC	Boster	BA1110	IF	1:60
Rabbit anti-goat IgG-Cy3	Boster	BA1034	IF	1:60
Goat anti-mouse IgG-Cy3	Boster	BA1031	IF	1:60
Goat anti-mouse IgG-FITC	Boster	BA1101	IF	1:60
Goat anti-rabbit IgG-Cy3	Boster	BA1032	IF	1:60
Donkey anti-mouse IgG (H + L)(Alexa Fluor 555)	Beyotime	A0460	IF	1:500
Anti-IdU	Novus	NBP2-44056	DNA fibre	1:300

### Cell proliferation assays

#### Growth curves

hDPCs were seeded in 96-well plates at a density of 3 × 10^3^ cells per well in triplicate for each time point, and the medium was changed every 2 days. To determine the cell viability, a 7-day time course was implemented, and a 10-μl CCK-8 solution (Dojindo) was added every 2 days and incubated for 1.5 h according to the manufacturer’s protocol. The absorbance at 450 nm was measured by a microplate reader (BioTek) and normalized to the background readings.

### Colony formation assay

hDPCs were seeded in a six-well plate at a density of 500 cells/well and cultured in growth medium for 3 wks. Cells were washed with PBS, fixed in a 4% paraformaldehyde for 5 min, and then stained with crystal violet (Beyotime) for 5 min.

### Flow cytometry analysis

To analyse the cell cycle distribution, trypsinized cells were washed with PBS and fixed in ice-cold 70% ethanol overnight. Cells were then washed 2X with PBS and incubated with RNase for 30 min at 37 °C followed by incubation with propidium iodide (PI) (KeyGEN Biotech) for 30 min at 4 °C. The PI-stained cells were examined on a Guava EasyCyte HT flow cytometer (Millipore) and analysed with InCyte2.7 software (Millipore).

### RNA-sequencing analysis

hDPCs from three different donors were collected, treated with TRIzol (Invitrogen) and subjected to cell cycle RNA sequencing (RiboBio) to detect the transcripts of cell cycle-related genes. Array and data were performed and analysed by RiboBio Inc. (Guangzhou). Genes with an expression fold change ≥ 1.5 and *P* ≤ 0.05 were considered differentially expressed. RNA-Seq data are available upon demand from the corresponding author.

### qPCR

Total RNA was extracted using TRIzol (Invitrogen) according to the manufacturer’s protocol. Reverse transcription was performed with a PrimeScript^®^ RT reagent kit with gDNA Eraser (TaKaRa). Quantitative real-time PCR was carried out using a standard SYBR Green PCR kit (TaKaRa) on a CFX96 (Bio-Rad). Glyceraldehyde-3-phosphate dehydrogenase (*GAPDH*) was used to normalize the expression level of each gene. Primer pairs for each gene were as follows:

- *GAPDH*: 5′-CGGAGTCAACGGATTTGGTCGTAT-3′ and 5′-AGCCTTCTCCATGGTGGTGAAGAC-3′;
- EZH2: 5′-GCCAGACTGGGAAGAAATCTG-3′ and 5′-TGTGCTGGAAAATCCAAGTCA-3′;
- *CDK2*: 5′-GGCACGTACGGAGTTGTGTA-3′ and 5′-CTCAGTCTCAGTGTCCAGGC-3′;
- *ANAPC1*: 5′-AGGCCTGCGAAGGAAACTTA-3′ and 5′-ACGTTGACACGGACAGGATG-3′;
- *CDKN2B*: 5′-GAATGCGCGAGGAGAACAAG-3′ and 5′-CATCATCATGACCTGGATCGC-3′;
- *SMAD2*: 5′-GCTTCCCTCGTGCTGATTGG-3′ and 5′-GTATGGAAGACGGAGGGAGC-3′;
- *CCNE2*: 5′-GGCCTATATATTGGGTTGGCG-3′ and 5′-ACGGCTACTTCGTCTTGACA-3′;
- *MCM2*: 5′-ATCTACGCCAAGGAGAGGGT-3′ and 5′-GTAATGGGGATGCTGCCTGT-3′;
- *BUB1B*: 5′-GGATGGGTCCTTCTGGAAAC-3′ and 5′-AAGCTCCCCAAGAACAGACA-3′;
- *CDC27*: 5′-GACAGCTCCACAACCAAGGA-3′ and 5′-TCAAACCTTCTGCTGCTGCT-3′.

### Immunofluorescence staining

hDPCs were seeded on coverslips overnight before serum deprivation. Cells were fixed with 4% paraformaldehyde in PBS at room temperature for 15 min and then permeabilized with 0.5% Triton X-100 in PBS for 20 min at room temperature. Cells were blocked for 30 min with blocking buffer (Zhongshanjinqiao) and incubated overnight at 4 °C with primary antibodies in blocking solution. Coverslips were washed 5X with PBST (0.5% Tween-20) and incubated for 1 h at room temperature with fluorescent secondary antibodies (Table 1). Next, the coverslips were washed 5X with PBST (0.5% Tween-20) and then stained with 4′6-diamidino-2-phenylindole (DAPI) to identify nuclear DNA. Images were captured on a Nikon Eclipse 300 fluorescence microscope (CompixInc). ImageJ was used to calculate Pearson’s correlation coefficient and Mender’s coefficient of colocalization.

### 5-Ethynyl-2′-deoxyuridine (EdU) staining

EdU staining was conducted using an EdU imaging kit (RiboBio) according to the manufacturer’s protocol. Briefly, hDPCs were seeded on coverslips overnight followed by serum starvation. Then, cells were released from deprivation in complete growth medium for 24 h, during which an EdU pulse was applied for 3 h at a concentration of 50 μM. After labelling, cells were stained with EdU. For double staining with other antigens, additional immunohistochemical staining was performed following EdU staining before the nuclei were stained with DAPI. Images were captured with a Nikon Eclipse 300 fluorescence microscope (CompixInc).

### Proximity ligation assay

Cells were prepared as described in the immunofluorescence section and incubated with anti-EZH2 (Goat) and anti-PCNA (mouse) primary antibodies following the manufacturer’s instructions (Sigma-Aldrich). Briefly, cells were incubated for 60 min at 37 °C with anti-mouse PLUS (Sigma-Aldrich, DUO92001) and anti-Goat MINUS (Sigma-Aldrich, DUO92006), washed twice with Buffer A (Sigma-Aldrich, DUO82047), and then incubated with the ligation solution (Sigma-Aldrich, DUO92014) for 30 min at 37 °C. After ligation, cells were washed twice with Buffer A and then incubated for 100 min at 37 °C with amplification reagents (Sigma-Aldrich, DUO92014). Finally, the cells were washed three times with Buffer B (Sigma-Aldrich, DUO82048), stained, and mounted with mounting medium (Sigma-Aldrich, DUO82040) to visualize the nuclei. Images were captured with a Nikon Eclipse 300 fluorescence microscope (CompixInc).

### Immunoprecipitation

Cells were harvested at the indicated times, lysed in lysis buffer supplemented with a protease inhibitor cocktail, incubated on ice for 20 min, and cleared by centrifugation at 14,000 rpm at 4 °C for 20 min. Total protein lysate (500 μg) was incubated with agarose-conjugated protein A/G beads for 2 h at 4 °C; the lysate was washed 5X with PBS at 4 °C and centrifuged at 14,000 rpm at 4 °C for 20 min. Then, the lysate was subjected to immunoprecipitation with agarose (Beyotime)-immobilized antibodies or isotype control antibodies overnight at 4 °C. The mix was washed 3X with lysis buffer at 4 °C. The precipitated proteins were subjected to SDS-PAGE and detected by western blot.

### Formaldehyde crosslinking

Formaldehyde crosslinking assays were performed as previously described [65]. Trypsinized cells were resuspended and incubated in a 1.0–1.5% formaldehyde solution at room temperature for 30 min. Then, the reaction was quenched by the addition of a 1.5 M glycine solution to a final concentration of 0.15 M and incubated at room temperature for 5 min. Cells were pelleted by centrifugation at 4000 rpm at room temperature for 5 min and resuspended and washed twice in PBS. Products were analysed by western blot using PCNA (PC10) or Myc-tagged antibodies.

### DNA fibre assay

A DNA fibre assay was performed according to a previous study [66]. Briefly, 293T cells were labelled with 25 μM IdU (Sigma-Aldrich, I17125) for 2 h. A 2-μl cell suspension was spotted at the end of the microscope slide and incubated with 7 μl of lysis buffer (0.5% SDS, 200 mM Tris–HCl (pH 7.4), and 50 mM EDTA) for 2 min. Slides were tilted 15° to allow the DNA fibres to stretch along the slide and then allowed to air-dry. After 10 min of fixation in methanol:acetic acid (3:1), the DNA was denatured with 2.5 M HCl for 60 min and blocked in 5% BSA in PBS (Table 1).

Slides were incubated with primary antibodies (anti-IdU), washed three times in PBS, incubated with secondary antibodies, and mounted.

### Statistical analysis

The statistical analysis of the RNA sequencing was calculated with ANOVA, whereas the statistical analysis of other experiments was carried out with Student’s *t test* (two-tailed) using Prism 5 (GraphPad Software). Error bars represent the standard deviation (SD). *P* values < 0.05 were considered statistically significant. *P < 0.05, **P < 0.01, and ***P < 0.001. All experiments were repeated three times biologically unless specifically indicated.

## Additional files

**Additional file 1: Figure S1 (related to Fig.** **1).** A: Immunoblot shows the H3K27me3 level in EZH2-knockdown hDPCs. B: The morphology of hDPCs with EZH2 knockdown. C: Quantification of colony-forming ability of hDPCs with EZH2 knockdown. D: Charts represent genes in the cell cycle RNA-sequencing assay that are involved in DNA replication based on the AmiGO 2 database. (*ns* not significant, *P < 0.05, **P < 0.01, ***P < 0.001). Scale bar represents 100 μm

**Additional file 2: Figure S2 (related to Fig.** **2).** A: Flow cytometry shows the cell cycle distribution of hDPCs from one donor at the indicated time points after release from serum deprivation. B: Immunoblot demonstrates the expression of CYCLIN D1 and CYCLIN A2, indicating G1 phase and S phase, respectively. α-TUBULIN was used a loading control of whole lysate. C: Statistical analysis of the EZH2-positive ratio (Left, *n* = 3) and Pearson’s correlation coefficient of EZH2 with DAPI (right, each dot represents one cell). D: Statistical analysis of the PLA-positive ratio (left, *n* = 5). The PLA signals are associated with DAPI on a single-cell basis (***P < 0.001)

**Additional file 3: Figure S3.** Uncut immunoblots from the immunoprecipitation experiments

## References

[1] Barretina J, Caponigro G, Stransky N, Venkatesan K, Margolin AA, Kim S, Wilson CJ, Lehar J, Kryukov GV, Sonkin D (2012). The cancer cell line encyclopedia enables predictive modelling of anticancer drug sensitivity. Nature.

[2] Casimiro MC, Crosariol M, Loro E, Li Z, Pestell RG (2012). Cyclins and cell cycle control in cancer and disease. Genes Cancer.

[3] Champeris Tsaniras S, Kanellakis N, Symeonidou IE, Nikolopoulou P, Lygerou Z, Taraviras S (2014). Licensing of DNA replication, cancer, pluripotency and differentiation: an interlinked world?. Semin Cell Dev Biol.

[4] Moldovan GL, Pfander B, Jentsch S (2007). PCNA, the maestro of the replication fork. Cell.

[5] Krishna TS, Fenyo D, Kong XP, Gary S, Chait BT, Burgers P, Kuriyan J (1994). Crystallization of proliferating cell nuclear antigen (PCNA) from *Saccharomyces cerevisiae*. J Mol Biol.

[6] Krishna TS, Kong XP, Gary S, Burgers PM, Kuriyan J (1994). Crystal structure of the eukaryotic DNA polymerase processivity factor PCNA. Cell.

[7] Kong XP, Onrust R, O’Donnell M, Kuriyan J (1992). Three-dimensional structure of the beta subunit of *E. coli* DNA polymerase III holoenzyme: a sliding DNA clamp. Cell.

[8] Hoege C, Pfander B, Moldovan GL, Pyrowolakis G, Jentsch S (2002). RAD6-dependent DNA repair is linked to modification of PCNA by ubiquitin and SUMO. Nature.

[9] Mailand N, Gibbs-Seymour I, Bekker-Jensen S (2013). Regulation of PCNA-protein interactions for genome stability. Nat Rev Mol Cell Biol.

[10] Watanabe K, Tateishi S, Kawasuji M, Tsurimoto T, Inoue H, Yamaizumi M (2004). Rad18 guides pol eta to replication stalling sites through physical interaction and PCNA monoubiquitination. EMBO J.

[11] Hasan S, Hassa PO, Imhof R, Hottiger MO (2001). Transcription coactivator p300 binds PCNA and may have a role in DNA repair synthesis. Nature.

[12] Jorgensen S, Elvers I, Trelle MB, Menzel T, Eskildsen M, Jensen ON, Helleday T, Helin K, Sorensen CS (2007). The histone methyltransferase SET8 is required for S-phase progression. J Cell Biol.

[13] Takawa M, Cho HS, Hayami S, Toyokawa G, Kogure M, Yamane Y, Iwai Y, Maejima K, Ueda K, Masuda A (2012). Histone lysine methyltransferase SETD8 promotes carcinogenesis by deregulating PCNA expression. Cancer Res.

[14] Greer EL, Shi Y (2012). Histone methylation: a dynamic mark in health, disease and inheritance. Nat Rev Genet.

[15] Black JC, Van Rechem C, Whetstine JR (2012). Histone lysine methylation dynamics: establishment, regulation, and biological impact. Mol Cell.

[16] Biggar KK, Li SS (2015). Non-histone protein methylation as a regulator of cellular signalling and function. Nat Rev Mol Cell Biol.

[17] Hamamoto R, Saloura V, Nakamura Y (2015). Critical roles of non-histone protein lysine methylation in human tumorigenesis. Nat Rev Cancer.

[18] Czermin B, Melfi R, McCabe D, Seitz V, Imhof A, Pirrotta V (2002). Drosophila enhancer of Zeste/ESC complexes have a histone H3 methyltransferase activity that marks chromosomal Polycomb sites. Cell.

[19] Cao R, Wang L, Wang H, Xia L, Erdjument-Bromage H, Tempst P, Jones RS, Zhang Y (2002). Role of histone H3 lysine 27 methylation in Polycomb-group silencing. Science.

[20] Kuzmichev A, Nishioka K, Erdjument-Bromage H, Tempst P, Reinberg D (2002). Histone methyltransferase activity associated with a human multiprotein complex containing the Enhancer of Zeste protein. Genes Dev.

[21] Cao R, Zhang Y (2004). The functions of E(Z)/EZH2-mediated methylation of lysine 27 in histone H3. Curr Opin Genet Dev.

[22] Lee JM, Lee JS, Kim H, Kim K, Park H, Kim JY, Lee SH, Kim IS, Kim J, Lee M (2012). EZH2 generates a methyl degron that is recognized by the DCAF1/DDB1/CUL4 E3 ubiquitin ligase complex. Mol Cell.

[23] Kim E, Kim M, Woo DH, Shin Y, Shin J, Chang N, Oh YT, Kim H, Rheey J, Nakano I (2013). Phosphorylation of EZH2 activates STAT3 signaling via STAT3 methylation and promotes tumorigenicity of glioblastoma stem-like cells. Cancer Cell.

[24] He A, Shen X, Ma Q, Cao J, von Gise A, Zhou P, Wang G, Marquez VE, Orkin SH, Pu WT (2012). PRC2 directly methylates GATA4 and represses its transcriptional activity. Genes Dev.

[25] Varambally S, Dhanasekaran SM, Zhou M, Barrette TR, Kumar-Sinha C, Sanda MG, Ghosh D, Pienta KJ, Sewalt RG, Otte AP (2002). The polycomb group protein EZH2 is involved in progression of prostate cancer. Nature.

[26] Bracken AP, Pasini D, Capra M, Prosperini E, Colli E, Helin K (2003). EZH2 is downstream of the pRB-E2F pathway, essential for proliferation and amplified in cancer. EMBO J.

[27] Bracken AP, Kleine-Kohlbrecher D, Dietrich N, Pasini D, Gargiulo G, Beekman C, Theilgaard-Monch K, Minucci S, Porse BT, Marine JC (2007). The Polycomb group proteins bind throughout the INK4A-ARF locus and are disassociated in senescent cells. Genes Dev.

[28] Chou DM, Adamson B, Dephoure NE, Tan X, Nottke AC, Hurov KE, Gygi SP, Colaiacovo MP, Elledge SJ (2010). A chromatin localization screen reveals poly (ADP ribose)-regulated recruitment of the repressive polycomb and NuRD complexes to sites of DNA damage. Proc Natl Acad Sci USA.

[29] Rondinelli B, Gogola E, Yucel H, Duarte AA, van de Ven M, van der Sluijs R, Konstantinopoulos PA, Jonkers J, Ceccaldi R, Rottenberg S (2017). EZH2 promotes degradation of stalled replication forks by recruiting MUS81 through histone H3 trimethylation. Nat Cell Biol.

[30] Piunti A, Rossi A, Cerutti A, Albert M, Jammula S, Scelfo A, Cedrone L, Fragola G, Olsson L, Koseki H, Testa G (2014). Polycomb proteins control proliferation and transformation independently of cell cycle checkpoints by regulating DNA replication. Nat Commun.

[31] Wu Z, Lee ST, Qiao Y, Li Z, Lee PL, Lee YJ, Jiang X, Tan J, Aau M, Lim CZ (2011). Polycomb protein EZH2 regulates cancer cell fate decision in response to DNA damage. Cell Death Differ.

[32] Gonzalez ME, Li X, Toy K, DuPrie M, Ventura AC, Banerjee M, Ljungman M, Merajver SD, Kleer CG (2009). Downregulation of EZH2 decreases growth of estrogen receptor-negative invasive breast carcinoma and requires BRCA1. Oncogene.

[33] Kotake Y, Cao R, Viatour P, Sage J, Zhang Y, Xiong Y (2007). pRB family proteins are required for H3K27 trimethylation and Polycomb repression complexes binding to and silencing p16INK4alpha tumor suppressor gene. Genes Dev.

[34] Hanahan D, Weinberg RA (2000). The hallmarks of cancer. Cell.

[35] Petruk S, Cai J, Sussman R, Sun G, Kovermann SK, Mariani SA, Calabretta B, McMahon SB, Brock HW, Iacovitti L (2017). Delayed Accumulation of H3K27me3 on nascent DNA is essential for recruitment of transcription factors at early stages of stem cell differentiation. Mol Cell.

[36] Petruk S, Sedkov Y, Johnston DM, Hodgson JW, Black KL, Kovermann SK, Beck S, Canaani E, Brock HW, Mazo A (2012). TrxG and PcG proteins but not methylated histones remain associated with DNA through replication. Cell.

[37] Bravo R, Frank R, Blundell PA, Macdonald-Bravo H (1987). Cyclin/PCNA is the auxiliary protein of DNA polymerase-delta. Nature.

[38] Hansen KH, Bracken AP, Pasini D, Dietrich N, Gehani SS, Monrad A, Rappsilber J, Lerdrup M, Helin K (2008). A model for transmission of the H3K27me3 epigenetic mark. Nat Cell Biol.

[39] Trievel RC, Beach BM, Dirk LM, Houtz RL, Hurley JH (2002). Structure and catalytic mechanism of a SET domain protein methyltransferase. Cell.

[40] Zhang X, Tamaru H, Khan SI, Horton JR, Keefe LJ, Selker EU, Cheng X (2002). Structure of the neurospora SET domain protein DIM-5, a histone H3 lysine methyltransferase. Cell.

[41] Dillon SC, Zhang X, Trievel RC, Cheng X (2005). The SET-domain protein superfamily: protein lysine methyltransferases. Genome Biol.

[42] Warbrick E (1998). PCNA binding through a conserved motif. BioEssays News Rev Mol Cell Dev Biol.

[43] Gilljam KM, Feyzi E, Aas PA, Sousa MM, Muller R, Vagbo CB, Catterall TC, Liabakk NB, Slupphaug G, Drablos F (2009). Identification of a novel, widespread, and functionally important PCNA-binding motif. J Cell Biol.

[44] Raynaud C, Sozzani R, Glab N, Domenichini S, Perennes C, Cella R, Kondorosi E, Bergounioux C (2006). Two cell-cycle regulated SET-domain proteins interact with proliferating cell nuclear antigen (PCNA) in arabidopsis. Plant J Cell Mol Biol.

[45] Kogure M, Takawa M, Saloura V, Sone K, Piao L, Ueda K, Ibrahim R, Tsunoda T, Sugiyama M, Atomi Y (2013). The oncogenic polycomb histone methyltransferase EZH2 methylates lysine 120 on histone H2B and competes ubiquitination. Neoplasia.

[46] Ducoux M, Urbach S, Baldacci G, Hubscher U, Koundrioukoff S, Christensen J, Hughes P (2001). Mediation of proliferating cell nuclear antigen (PCNA)-dependent DNA replication through a conserved p21(Cip1)-like PCNA-binding motif present in the third subunit of human DNA polymerase delta. J Biol Chem.

[47] Campbell S, Ismail IH, Young LC, Poirier GG, Hendzel MJ (2013). Polycomb repressive complex 2 contributes to DNA double-strand break repair. Cell Cycle.

[48] Bruning JB, Shamoo Y (2004). Structural and thermodynamic analysis of human PCNA with peptides derived from DNA polymerase-delta p66 subunit and flap endonuclease-1. Structure.

[49] Li H, Xie B, Zhou Y, Rahmeh A, Trusa S, Zhang S, Gao Y, Lee EY, Lee MY (2006). Functional roles of p12, the fourth subunit of human DNA polymerase delta. J Biol Chem.

[50] Gerik KJ, Li X, Pautz A, Burgers PM (1998). Characterization of the two small subunits of *Saccharomyces cerevisiae* DNA polymerase delta. J Biol Chem.

[51] Schuettengruber B, Chourrout D, Vervoort M, Leblanc B, Cavalli G (2007). Genome regulation by polycomb and trithorax proteins. Cell.

[52] Strahl BD, Allis CD (2000). The language of covalent histone modifications. Nature.

[53] Schuettengruber B, Bourbon HM, Di Croce L, Cavalli G (2017). Genome regulation by polycomb and trithorax: 70 years and counting. Cell.

[54] Shi Y, Lan F, Matson C, Mulligan P, Whetstine JR, Cole PA, Casero RA, Shi Y (2004). Histone demethylation mediated by the nuclear amine oxidase homolog LSD1. Cell.

[55] Warbrick E (1998). PCNA binding through a conserved motif. BioEssays News Rev Mol Cell Dev Biol.

[56] Jacob Y, Feng S, LeBlanc CA, Bernatavichute YV, Stroud H, Cokus S, Johnson LM, Pellegrini M, Jacobsen SE, Michaels SD (2009). ATXR5 and ATXR6 are H3K27 monomethyltransferases required for chromatin structure and gene silencing. Nat Struct Mol Biol.

[57] Chuang LSH, Ian HI, Koh TW, Ng HH, Xu GL, Li BFL (1997). Human DNA (cytosine-5) methyltransferase PCNA complex as a target for p21(WAF1). Science.

[58] Milutinovic S, Zhuang QL, Szyf M (2002). Proliferating cell nuclear antigen associates with histone deacetylase activity, integrating DNA replication and chromatin modification. J Biol Chem.

[59] Barrero MJ, Izpisua Belmonte JC (2013). Polycomb complex recruitment in pluripotent stem cells. Nat Cell Biol.

[60] Leung KHT, Abou El Hassan M, Bremner R (2013). A rapid and efficient method to purify proteins at replication forks under native conditions. BioTechniques.

[61] Kelman Z, O’Donnell M (1995). Structural and functional similarities of prokaryotic and eukaryotic DNA polymerase sliding clamps. Nucleic Acids Res.

[62] Smith BC, Denu JM (2009). Chemical mechanisms of histone lysine and arginine modifications. Biochem Biophys Acta.

[63] Garg P, Burgers PMJ (2005). DNA polymerases that propagate the eukaryotic DNA replication fork. Crit Rev Biochem Mol.

[64] Riva F, Savio M, Cazzalini O, Stivala LA, Scovassi IA, Cox LS, Ducommun B, Prosperi E (2004). Distinct pools of proliferating cell nuclear antigen associated to DNA replication sites interact with the p125 subunit of DNA polymerase delta or DNA ligase I. Exp Cell Res.

[65] Gronthos S, Mankani M, Brahim J, Robey PG, Shi S (2000). Postnatal human dental pulp stem cells (DPSCs) in vitro and in vivo. Proc Natl Acad Sci USA.

[66] Schwab RA, Niedzwiedz W (2011). Visualization of DNA replication in the vertebrate model system DT40 using the DNA fiber technique. J Vis Exp JoVE.

