# Supplementary figures and images for: EZH2 promotes DNA replication by stabilizing interaction of POLδ and PCNA via methylation-mediated PCNA trimerization
Source: Epigenetics Chromatin. 2018 Aug 2;11:44. doi: 10.1186/s13072-018-0213-1 (PMC6071395; doi:10.1186/s13072-018-0213-1)

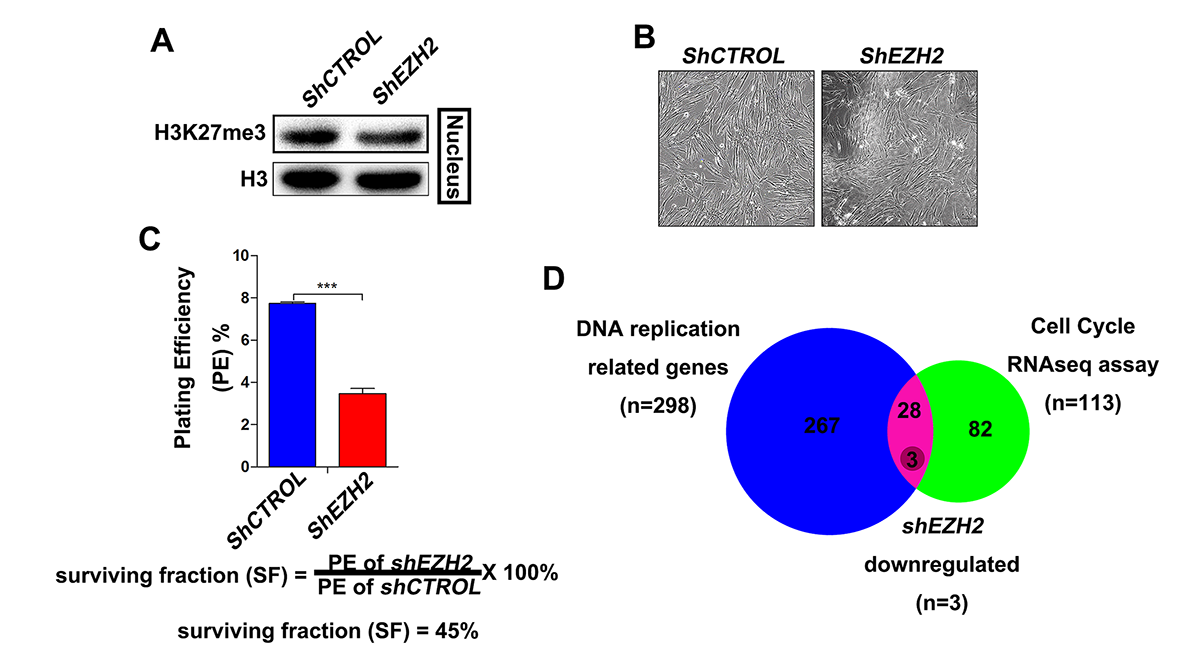

Supplement: Supplementary file 1 — Additional file 1: Figure S1 (related to Fig. 1). A: Immunoblot shows the H3K27me3 level in EZH2-knockdown hDPCs. B: The morphology of hDPCs with EZH2 knockdown. C: Quantification of colony-forming ability of hDPCs with EZH2 knockdown. D: Charts represent genes in the cell cycle RNA-sequencing assay that are involved in DNA replication based on the AmiGO 2 database. (ns not significant, *P < 0.05, **P < 0.01, ***P < 0.001). Scale bar represents 100 μm [file 13072_2018_213_MOESM1_ESM.tif]

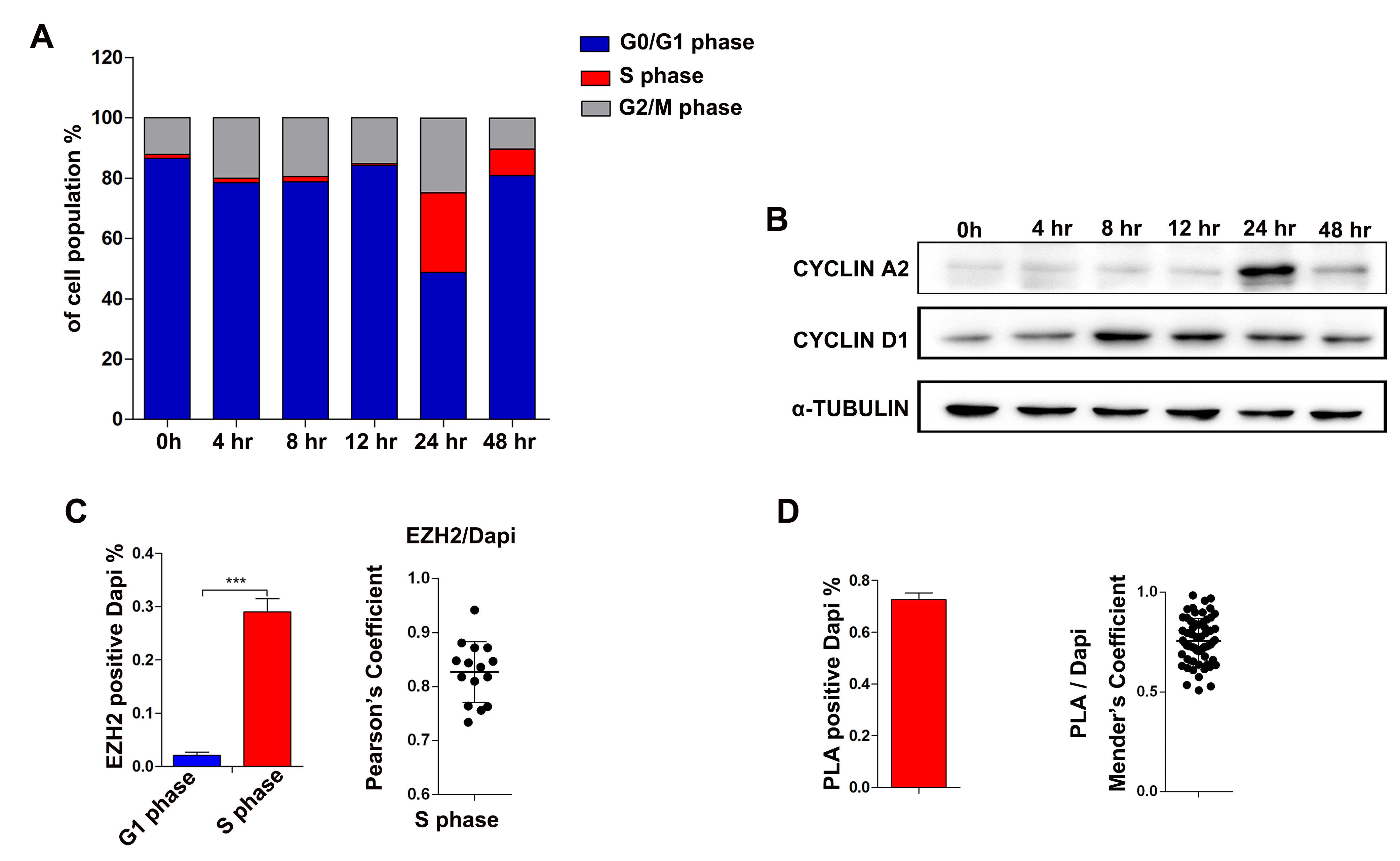

Supplement: Supplementary file 2 — Additional file 2: Figure S2 (related to Fig. 2). A: Flow cytometry shows the cell cycle distribution of hDPCs from one donor at the indicated time points after release from serum deprivation. B: Immunoblot demonstrates the expression of CYCLIN D1 and CYCLIN A2, indicating G1 phase and S phase, respectively. α-TUBULIN was used a loading control of whole lysate. C: Statistical analysis of the EZH2-positive ratio (Left, n = 3) and Pearson’s correlation coefficient of EZH2 with DAPI (right, each dot represents one cell). D: Statistical analysis of the PLA-positive ratio (left, n = 5). The PLA signals are associated with DAPI on a single-cell basis (***P < 0.001) [file 13072_2018_213_MOESM2_ESM.tif]

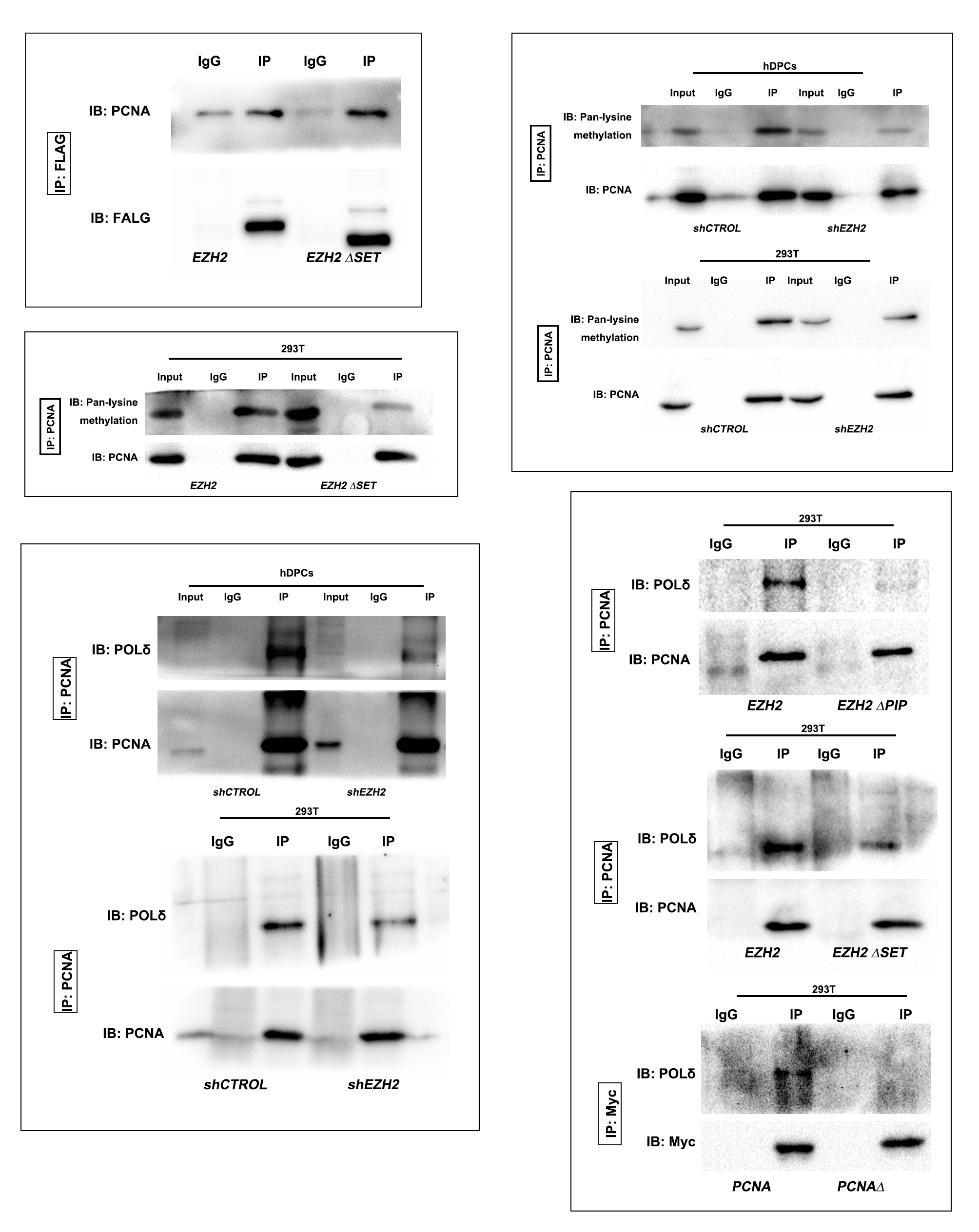

Supplement: Supplementary file 3 — Additional file 3: Figure S3. Uncut immunoblots from the immunoprecipitation experiments [file 13072_2018_213_MOESM3_ESM.tif]
